# Perineal Turn over Perforator Flap: A Novel Surgical Technique for Combined Perineal and Posterior Vaginal Wall Reconstruction

**DOI:** 10.29252/wjps.10.1.114

**Published:** 2021-01

**Authors:** Francisco S. Moura, Maria Chasapi, Peter Mitchell, Milind D. Dalal

**Affiliations:** 1 Department of Plastic Surgery - Royal Preston Hospital, Preston, UK; 2 Department of Colorectal Surgery - Royal Preston Hospital, Preston, UK

**Keywords:** Extralevator abdominoperineal excision, Fasciocutaneous, Internal pudendal artery, Perineum, Surgical reconstructive procedure surgery.

## Abstract

Extralevator Abdominoperineal Excision (ELAPE) and Abdominoperineal Resection create complex perineal defects made more challenging when combined with additional resection of the posterior vaginal wall. This composite defect requires the restoration of a functional vagina, in addition to the obliteration of the large perineal dead space, a need to reduce donor site, and perineal wound morbidity. Previously described fasciocutaneous and myocutaneous flaps for such defects are associated with long operations requiring intra-operative mobilization and are linked to post-operative complications including herniation, evisceration, flap loss, donor site morbidity and poor cosmetic outcome, amongst other issues. Herein we describe the case of a 60-year-old female patient that underwent combined ELAPE and posterior vaginectomy for anal squamous cell carcinoma. This complex defect was reconstructed using an extended version of the Perineal Turn-Over (PTO) flap based on the Internal Pudendal artery perforator.

## INTRODUCTION

**Fig. 1:**
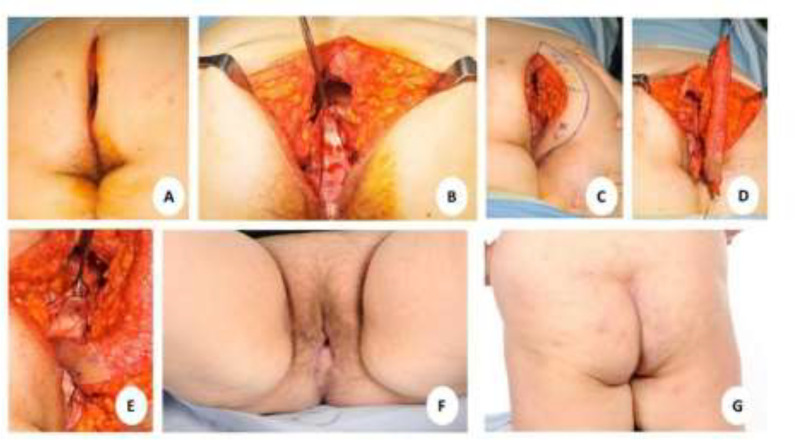
Key steps in combined posterior vaginal wall defect and perineal reconstruction post- Extralevator Abdominoperineal Excision (ELAPE) using Perineal Turnover (PTO) flap. **(a-b)** The patient in prone jack-knife position post-ELAPE with demonstration of the anterior vaginal wall. **(c)** A perforator of the Internal Pudendal artery is identified with a handheld Doppler ultrasound at the inferolateral part of the skin defect and a semilunar area of skin incorporating the perforator is marked. **(d)** The marked flap is incised down to and raised in the supra-fascial plane. **(e)** The inferior aspect of the flap is rotated inwards and the skin is sutured to the anterior vaginal wall. The thick gluteal dermis of the remaining flap is then sutured to the cut end of the pelvic wall muscles. **(f-g)** Results at six weeks post-operative

Abdominoperineal Resections (APR) and Extralevator Abdominoperineal Excision (ELAPE) carry a significant risk of perineal wound problems^1^. Both procedures can be used in the management of low rectal cancers with the latter becoming an increasingly popular technique in the surgical management of both low rectal and anal tumours. ELAPE has demonstrated reduced rates of bowel perforation and superior oncological outcomes leading to improved local disease and survival^1^^–^^4^. ELAPE refers to the En-bloc resection of the anorectum together with the Levator Ani muscles. This implies a challenging and larger extensive three-dimensional soft tissue perineal defect when compared to the more conventional APR^5^. Due to the proximity of vital pelvic structures to the rectal region, simultaneous resection of the posterior vaginal wall is commonly carried out resulting in an additional reconstructive challenge (Figure 1). 

We have previously reported that the Perineal Turn Over (PTO) flap can quickly, safely and reliably reconstruct post-ELAPE perineal defects whilst keeping donor site, perineal wound morbidity and perineal hernia rates low^6^. Herein we describe a case report where an extended version of this flap addresses the complex reconstructive dilemma of combined posterior vaginal wall and perineum defects post-ELAPE.

## CASE REPORT

A 60-year old woman was diagnosed with anal squamous cell carcinoma invading the PVW. She underwent pre-operative neo-adjuvant chemo-radiotherapy and a multidisciplinary decision was made to offer the patient ELAPE surgery. Under general anesthetic, the colorectal surgeons performed a laparoscopic-guided ELAPE in supine position and ended the procedure in a Jackknife position to complete the perineal element of the surgery. For the reconstructive operation, the patient remains in the same prone Jackknife position as in the final part of the ELAPE or APR procedure (Figure 1A). 

**Fig. 2:**
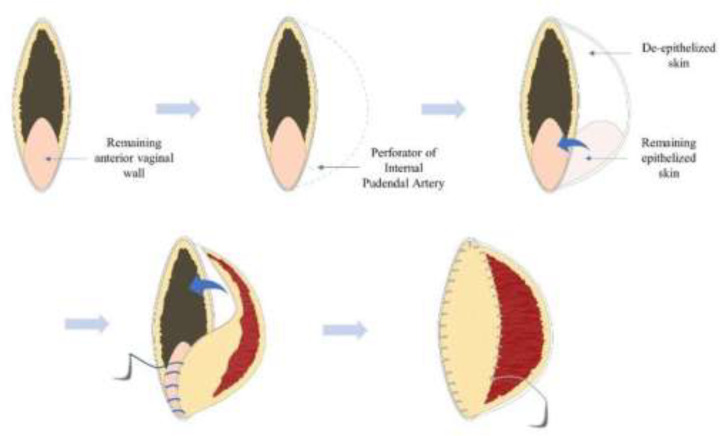
Visual illustration of the Perineal Turn Over (PTO) concept for reconstruction of perineal defect following extralevator abdominoperineal excision (ELAPE) and posterior vaginectomy

An Internal Pudendal artery perforator was identified with a handheld Doppler over the inferolateral aspect of the skin defect. A semilunar area of skin incorporating the perforator was marked along one side of the perineal defect (Figure 1C) where the width of the flap equals the width of the muscle defect in the perineal muscle floor. Skin on the flap superior to the perforator was de-epithelized as in the standard PTO flap^6^. Skin inferior to the perforator was not de-epithelialized. This skin, inferior to the perforator, was used to reconstruct the posterior vaginal wall. The marked skin island is incised down to the supra-fascial plane (Figure 1D). The inferior 5cm of skin paddle is folded inwards with the skin facing the anterior vaginal wall. This skin paddle is sutured to the remaining anterior vaginal wall to create sufficient vaginal volume to restore sexual function of the vagina. The superior 15 cm of the flap was de-epithelialized and turned inwards towards the perineal defect (Figures 1E and 2). The free border of the inverted thick gluteal dermis was sutured to the cut edges of the pelvic muscle using a parachute-like technique with a 2-0 PDS suture. The gluteal muscles are left undisturbed whilst the overlying gluteal skin on both buttocks was undermined and then advanced medially to be closed in layers over the midline using 3-0 PDS for subdermal and 3-0 Monocryl for subcuticular closure. This allowed for recreation of the natal cleft (Figure 1G). If deemed appropriate, a size ten Redivac drain may be inserted below the advanced gluteal skin flaps, although this was not used in this case.

Post-operatively, the patient was nursed on the opposite side of the flap side and was not allowed to sit and lie supine for two weeks to protect the perforator flap. Once the wounds healed, the patient was advised to use a vaginal dilator twice a day for four weeks post-operatively. The patient had a two-year follow-up with a satisfactory functional and aesthetic outcome. In addition, the patient did not experience postoperative complications. She was able to engage in sexual intercourse four months post-operatively.

Consent for publication: Relevant consent was obtained from the patient for medical illustration, publication and teaching purposes.

## DISCUSSION

This article describes a case report in which a surgical technique was used to reconstruct both a large central perineal and posterior partial vaginectomy defects. Cordeiro et al. classified an acquired posterior vaginal wall defect as 1B^7^. Fasciocutaneous and myocutaneous flaps are the most commonly reported options for reconstruction of such defects^8^. These reconstructive options are associated with important difficulties and complications in addition to requiring the expertise of a Plastic surgeon. Rectus abdominus flaps have reported risks of herniation, evisceration and flap loss secondary to ischemia whilst Singapore flaps can be complicated by apical necrosis as well as vulvar pain and hair growth. In addition, both these prolonged options need to be carried out in the lithotomy position requiring intra-operative change of position. The Singapore flap as well as the Gracilis flap have reported flap failure loss of up to 15%^9^^,^^10^. Superior and Inferior Gluteal artery perforator-based fasciocutaneous transposition flap was described to reconstruct perineal and partial vaginal wall defects^11^. Yet, this approach often requires transection of several muscle fibers for the flap to reach the perineal defect and to prevent the perforator from being stripped off by muscle contraction. 

To our knowledge, composite perineal and partial vaginal wall reconstruction following ELAPE and posterior vaginal wall resection is an unexplored area in current literature. Restoration of a functional vagina whilst reconstructing the perineal defect is a significant challenge. In addition, an optimum solution should avoid sacrifice of a functional muscle, not interfere with colostomy formation, and avoid using local irradiated tissue^11^.

Low donor site and perineal wound morbidity rates along with short operating time have made PTO flap the workhorse flap in our unit for post-ELAPE perineal defects, and post-APR when appropriate. Patients often have preoperative radiotherapy. A retrospective review of these patients’ radiotherapy CT scans with our clinical oncologist colleagues has shown that Internal Pudendal artery perforators are consistently outside the irradiation zone, confirming the safety profile of this flap. 

An extended version of the PTO flap herein presented offers a quick (operating time of 60 min), simple and safe solution for composite post-ELAPE perineal and PWW defects, adding to its versatility^12^^,^^13^. A further case series using a minimally modified similar technique has since been reported highlighting the benefits of this technique^14^. Despite the description of a single case, we look forward to building experience and data on this technique for this composite defect to confirm its various advantages. 

## CONCLUSION

The extended PTO flap provides the following benefits: 

1. The posterior vaginal wall is reconstructed with a skin flap allowing for functional restoration of the vagina. 

2. This procedure is carried out in prone position preventing further intra-operative change of position.

3. The donor site does not interfere with stoma formation.

4. The perforator based on the Internal Pudendal artery is usually protected from radiotherapy allowing for reliable vascularity.

5.The thick gluteal dermis acts as an autologous dermal vascularised substitute strengthening the pelvic floor and reducing the risk of herniation.

6. The gluteal subcutaneous flap obliterates the large dead space preventing fluid collections and subsequent infections and wound dehiscence. 

7. The final suture line of the natal cleft provides a good aesthetic outcome. 
